# The mechanisms of Ca^2+^ regulating autophagy and its research progress in neurodegenerative diseases: A review

**DOI:** 10.1097/MD.0000000000039405

**Published:** 2024-08-23

**Authors:** Meng Hou, Zhixiao Zhang, Zexin Fan, Lei Huang, Li Wang

**Affiliations:** a Department of Neurology, Second Hospital of Shanxi Medical University, Taiyuan, Shanxi, China; b Department of Neurology, Shanxi Provincial People’s Hospital, Taiyuan, Shanxi, China; c Department of Cardiology, Second Hospital of Shanxi Medical University, Taiyuan, Shanxi, China.

**Keywords:** autophagy, Ca^2+^, Ca^2+^ channels, Ca^2+^ signal transduction, Ca^2+^ storage compartments, neurodegenerative diseases

## Abstract

Neurodegenerative diseases are complex disorders that significantly challenge human health, with their incidence increasing with age. A key pathological feature of these diseases is the accumulation of misfolded proteins. The underlying mechanisms involve an imbalance in calcium homeostasis and disturbances in autophagy, indicating a likely correlation between them. As the most important second messenger, Ca^2+^ plays a vital role in regulating various cell activities, including autophagy. Different organelles within cells serve as Ca^2+^ storage chambers and regulate Ca^2+^ levels under different conditions. Ca^2+^ in these compartments can affect autophagy via Ca^2+^ channels or other related signaling proteins. Researchers propose that Ca^2+^ regulates autophagy through distinct signal transduction mechanisms, under normal or stressful conditions, and thereby contributing to the occurrence and development of neurodegenerative diseases. This review provides a systematic examination of the regulatory mechanisms of Ca^2+^ in cell membranes and different organelles, as well as its downstream pathways that influence autophagy and its implications for neurodegenerative diseases. This comprehensive analysis may facilitate the development of new drugs and provide more precise treatments for neurodegenerative diseases.

## 1. Introduction

As we age, the central nervous system gradually degenerates. Alzheimer disease (AD) and Parkinson disease (PD) are the most prevalent neurodegenerative diseases, with the majority being sporadic cases that typically onset later in life, mostly affecting individuals aged 60 or older. Approximately 10% of these cases are familial characterized by early onset and generally observed in individuals under 50 years old.^[1]^ Other neurodegenerative diseases include Huntington disease (HD) and amyotrophic lateral sclerosis (ALS). A significant pathological feature of most neurodegenerative diseases is the presence of abnormal misfolded proteins, such as β-amyloid, tau protein, mutant α-synuclein (α-syn), ahd Huntingtin (Htt) protein. These abnormal misfolded proteins exert toxic effects that lead to neuronal death and associated clinical symptoms. Autophagy, a crucial cellular process, plays a key role in the removal of these misfolded proteins.

An imbalance in calcium homeostasis, a significant pathogenic factor in neurodegenerative diseases, is involved in the regulation of autophagy. Recent studies have increasingly focused on how Ca^2+^ regulates autophagy in the context of these diseases, highlighting its extensive research and application potential in the field of neurodegenerative diseases.

## 2. Ca^2+^ and neurodegenerative diseases

As a second messenger, Ca^2+^ plays a critical role in various cellular processes. Neurons are particularly sensitive to even minor disturbances in intracellular Ca^2+^ levels. Ca^2+^ signaling regulates mitochondrial function and ATP production, which in turn affects synaptic transmission, learning, and memory formation. Calcium homeostasis is crucial for neuronal survival and has a significant impact on neurodegenerative diseases.^[2–4]^ In AD, elevated Ca^2+^ levels due to imbalances in calcium homeostasis can exacerbate the condition by promoting the production and aggregation of β-amyloid and phosphorylated Tau proteins.^[1]^ Mutations in presenilin disrupt calcium homeostasis, leading to altered processing of amyloid precursor protein (APP) and increased formation of amyloid β (Aβ), which further disrupts calcium homeostasis.^[5]^ In addition, dysregulation of Ca^2+^ contributes to synaptic loss, impacting learning and memory functions in AD patients.^[6,7]^ In PD, imbalances in calcium homeostasis can lead to α-syn aggregation and the formation of Lewy bodies, ultimately causing neuronal loss.^[8]^ In HD, cytoplasmic and mitochondrial Ca^2+^ overload can trigger apoptosis and neuronal degeneration, suggesting that Ca^2+^ channel blockers might be beneficial for treatment.^[9]^ Elevated Ca^2+^ levels have been observed in the spinal cords of mSOD1 mice used as models for ALS. Biophysical experiments have demonstrated that Ca^2+^-induced conformational changes in SOD1 contribute to its amorphous aggregation, a hallmark of ALS.^[10]^ These findings illustrate the effect of various misfolded proteins mediated by calcium homeostasis imbalance on various neurodegenerative diseases.

## 3. Ca^2+^ and autophagy

Autophagy plays a crucial role in the degradation and recovery of cellular components and is a conserved lysosomal degradation pathway present from yeast to humans. There are at least 3 types of autophagy: microautophagy, chaperone-mediated autophagy, and macroautophagy. Among these, macroautophagy, also known as the autolysosomal pathway, is the only autophagy pathway currently known to be regulated by Ca^2+^. During macroautophagy, a double-membrane vesicle structure is formed, which then expands, fuses, and engulfs cytoplasmic components to form autophagosomes. Abnormal autophagy has been identified as a significant mechanism of protein aggregation and deposition in various neurodegenerative diseases.^[11,12]^

Intracellular Ca^2+^ is a key regulator of autophagy. Buffering cytoplasmic calcium ([Ca^2+^]_C_) blocks the induction of autophagy, highlighting the crucial role of Ca^2+^ in this process. In 1993, Seglen et al provided the first evidence linking autophagy degradation with intracellular calcium homeostasis, demonstrating that Ca^2+^ in unknown cell compartments was essential for the autolysosomal degradation of proteins. They also noted the complex effects of Ca^2+^ on autophagy, as both increases and decreases in [Ca^2+^]_C_ could inhibit autophagy.^[13]^ Thus, the role of Ca^2+^ in autophagy remains controversial, with evidence suggesting it may have a dual effect. In the next 20 years, numerous studies both domestically and internationally have identified a variety of Ca^2+^ signaling-related proteins in the autophagy molecular network. These include Ca^2+^ channels in the plasma membrane, various Ca^2+^ storage compartments, as well as Ca^2+^-dependent proteases, which are distributed in different cellular regions. The imbalance of calcium homeostasis and the impairment of autophagy, as central pathogenesis in neurodegenerative diseases, are usually related.^[1]^ A systematic review of Ca^2+^ storage chambers and Ca^2+^ channels reveals their role in regulating calcium homeostasis and autophagy, thus influencing the occurrence and development of various neurodegenerative diseases. This understanding is crucial for identifying new therapeutic targets.

## 4. Effect of Ca^2+^ in different compartments on autophagy

**Figure 1.**
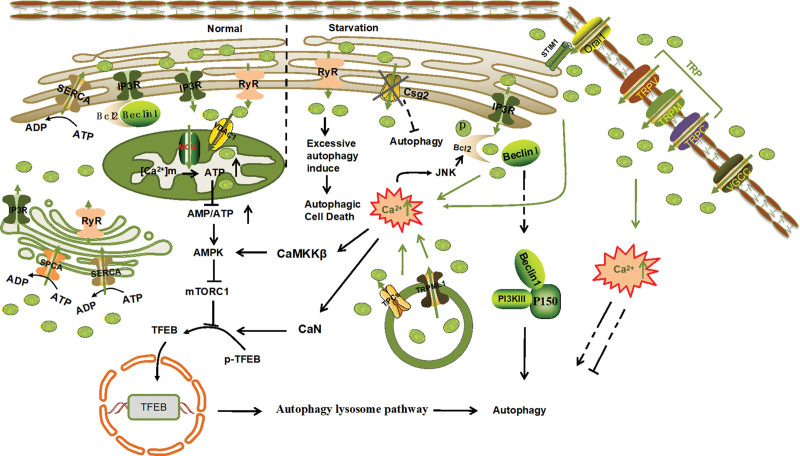
Mechanism diagram of Ca^2+^-regulating autophagy in different organelles. The calcium channel TRP on the cell membrane regulates autophagy through diverse mechanisms, either inducing or inhibiting the process. An elevation in mitochondrial Ca^2+^ influx boosts ATP production, enhances the AMPK/mTOR pathway, and consequently suppresses autophagy. During endoplasmic reticulum stress, the ER-located STIM1 protein binds to the Ca^2+^ channel Orai1 on the cell membrane, initiating causes Ca^2+^ influx and activating downstream signaling cascades to modulate autophagy. Overactivation of RyR channels elevates cytoplasmic Ca^2+^ levels, ultimately inducing autophagic cell death. Bcl-2 impacts autophagy by regulating the Beclin-1-IP3R-Bcl-2 complex, and an increase in intracellular Ca^2+^ levels can facilitate autophagy by activating JNK, which dissociates Beclin-1 from the Beclin-1-Bcl-2 complex. Furthermore, the closure of the Csg2 channel inhibits autophagy. Lysosomes release Ca^2+^ through TPC and TRPML1 channels to enhance cytoplasmic Ca^2+^ levels and activate downstream pathways to promote autophagy. There are also several Ca^2+^ channels in the Golgi apparatus, but the specific mechanisms by which they regulate autophagy remain unclear. AMPK = adenosine 5′-monophosphate (AMP)-activated protein kinase; Bcl-2 = B-cell lymphoma/leukemia 2; CaMKKβ = Ca^2+^-calmodulin-dependent kinase kinase β; CaN = calcineurin; IP3 = inositol-1,4,5-triphosphate; IP3R = IP3 receptors; JNK = c-Jun N-terminal kinase; MCU = mitochondrial Ca^2+^ uniporter; mTOR = mammalian target of rapamycin; Orai1 = Orai calcium Release-Activated calcium Modulator 1; RYR = ryanodine receptor; SERCA = The Sarco Endoplasmic Reticulum calcium ATPase; SPCA = secretory pathway Ca^2+^-ATPase; STIM1 = stromal interaction molecule; TFEB = transcription factor EB; TPCs = two-pore Ca^2+^ tunnels; TRP = transient receptor potential; TRPML1 = transient receptor potential melastatine 1; VDAC1 = the voltage-dependent anion channel 1; VGCC = voltage-gated calcium channel.

Intracellular Ca^2+^ is stored in various organelles and can be released into the cytoplasm to meet increasing physiological demands. Ca^2+^ in different compartments affects autophagy by regulating intracellular calcium homeostasis (Fig. 1).

### 4.1. Endoplasmic reticulum Ca^2+^ regulates autophagy

#### 4.1.1. Endoplasmic reticulum Ca^2+^ directly regulates autophagy

Research has found that inhibiting Ca^2+^ transients/Ca^2+^ oscillations on the surface of the endoplasmic reticulum (ER) can hinder the formation of autophagosomes. In cells with Ca^2+^ channel release inhibitors and knockdown of Ca^2+^ channel proteins, the aggregation of ER significantly inhibited the formation of the autophagy initiation complex FIP200, indicating that Ca^2+^ transients on the surface of ER is crucial for the formation of autophagosomes.^[14]^ The ER calcium ion concentration ([Ca^2+^]_ER_) is determined by 2 opposing processes: the activity of the Sarco/Endoplasmic Reticulum Ca^2+^ ATPase, which pumps calcium ions into the ER, and the release of Ca^2+^ from the ER through inositol-1,4,5-triphosphate and ryanodine receptor (RyR) channels.

In cells experiencing ER stress, elevated levels of [Ca^2+^]_C_ can activate various effector molecules that subsequently promote autophagy.^[11]^ This may occur because ER stress can block the interaction between inositol trisphosphate receptor (IP3R) and Beclin-1, allowing [Ca^2+^]_ER_ to be released into the cytoplasm.^[15]^ B-cell lymphoma/leukemia 2 (Bcl-2) is an antiapoptotic protein mainly located in the mitochondria, the ER, and the nuclear membrane, has dual effects on autophagy, either inhibiting or enhancing it. These contrasting actions might result from posttranslational modifications or different subcellular localizations of Bcl-2.^[16]^ So, how does Bcl-2 located in the ER affect ER calcium homeostasis? Studies have shown that Bcl-2 can inhibit the release of ER Ca^2+^ into the cytoplasm by enhancing the interaction between the IP3R-Beclin-1 complex.^[17]^ Down-regulation of Bcl-2 family proteins can relieve Beclin-1 from the IP3R-Beclin-1 complex, thereby promoting the formation of autophagosomes and ultimately enhancing protective autophagy.^[18]^ Increased [Ca^2+^]_C_ can activate c-Jun N-terminal kinase, leading to the dissociation of Beclin-1 from the Beclin-1-Bcl-2 complex. This process promotes autophagy and induces cytotoxicity in endothelial cells.^[19]^ Researches in human osteoarthritis and rat chondrocytes have confirmed the existence of the Beclin-1-IP3R-Bcl-2 complex within the ER, demonstrating its inhibitory effect on autophagy.^[20]^ Therefore, Bcl-2 may influence [Ca^2+^]_ER_ release through this complex, thereby regulating autophagy.

Insulin withdrawal leads to increased expression and activation of ryanodine receptor 3 (RyR3), which subsequently results in the release of Ca^2+^ from the ER. The increase in cytosolic Ca^2+^ subsequently triggers a signaling cascade that induces autophagic cell death (ACD) in HCN cells.^[21,22]^ Furthermore, the deletion of the ER calcium channel Csg2 causes elevated levels of ER Ca^2+^, resulting in the accumulation of sphingosine in bioactive sphingoid plants, which specifically blocks autophagy.^[23,24]^ Vacuole membrane protein 1 (VMP1) is an essential factor for Ca^2+^ release from the ER during homeostasis and has recently been identified as a crucial autophagy-related protein. The expression of VMP1 can be induced by starvation or rapamycin, which triggers the conversion of microtubule-associated protein 1 light chain 3 (LC3)-I to LC3-II, facilitating autophagosome formation in mammalian cells.^[25]^ Therefore, VMP1 may regulate autophagy by maintaining ER calcium homeostasis, although this relationship has not been fully confirmed.

#### 4.1.2. Endoplasmic reticulum Ca^2+^ indirectly regulates autophagy

The plasma membrane calcium channel N-methyl-D-aspartate receptor (NMDAR) facilitates the influx of extracellular Ca^2+^ into the intracellular space. This entry of Ca^2+^ triggers a response that is amplified through calcium-induced calcium release. The subsequent activation of IP3R leads to additional Ca^2+^ release and the activation of calmodulin (CaM). CaM may be activated either directly within the nucleus or, if activated in the cytosol, it translocates to the nucleus to activate calcium/CaM-dependent protein kinases.^[26]^ These kinases are closely related to autophagy.

The interaction between the ER and mitochondria occurs at specialized regions known as mitochondria-associated membranes, also referred to as mitochondria-ER contact sites. Ca^2+^ in the ER can indirectly affect autophagy by affecting mitochondrial functions. Under normal conditions, the effective transfer of Ca^2+^ between the ER and mitochondria is mediated by IP3R and RYR located in MAMs.^[27]^ Recently, a new family of TRPM8 channel isoforms has been identified as functional ER Ca^2+^ release channels expressed in MAMs.^[28]^ Increased mitochondrial calcium concentration ([Ca^2+^]_m_) promotes the mitochondrial tricarboxylic acid cycle and ATP production, which lowers the AMP/ATP ratio and inhibits AMP-activated protein kinase (AMPK) activity. This, in turn, stimulates the activity of mTORC1 and suppresses autophagy.^[11]^

IP3R and the voltage-dependent anion channel 1 (VDAC1) form a functional connection that facilitates the transfer of Ca^2+^ from the ER to the mitochondria. This interaction is mediated by Mortalin (also known as glucose-regulated protein 75), which acts as a connector protein between IP3R and VDAC1. Deletion of the endoplasmic reticulum channel IP3R leads to reduced mitochondrial Ca^2+^ uptake and a loss of bioenergy coupling.^[29]^ Knockdown of glucose-regulated protein 75 diminishes the interaction between IP3R and VDAC1 and attenuates cadmium-induced mitochondrial Ca^2+^ uptake in neuronal cells, as well as inhibiting cadmium-induced autophagy in PC12 cells and primary neurons.^[30]^

**Figure 2.**
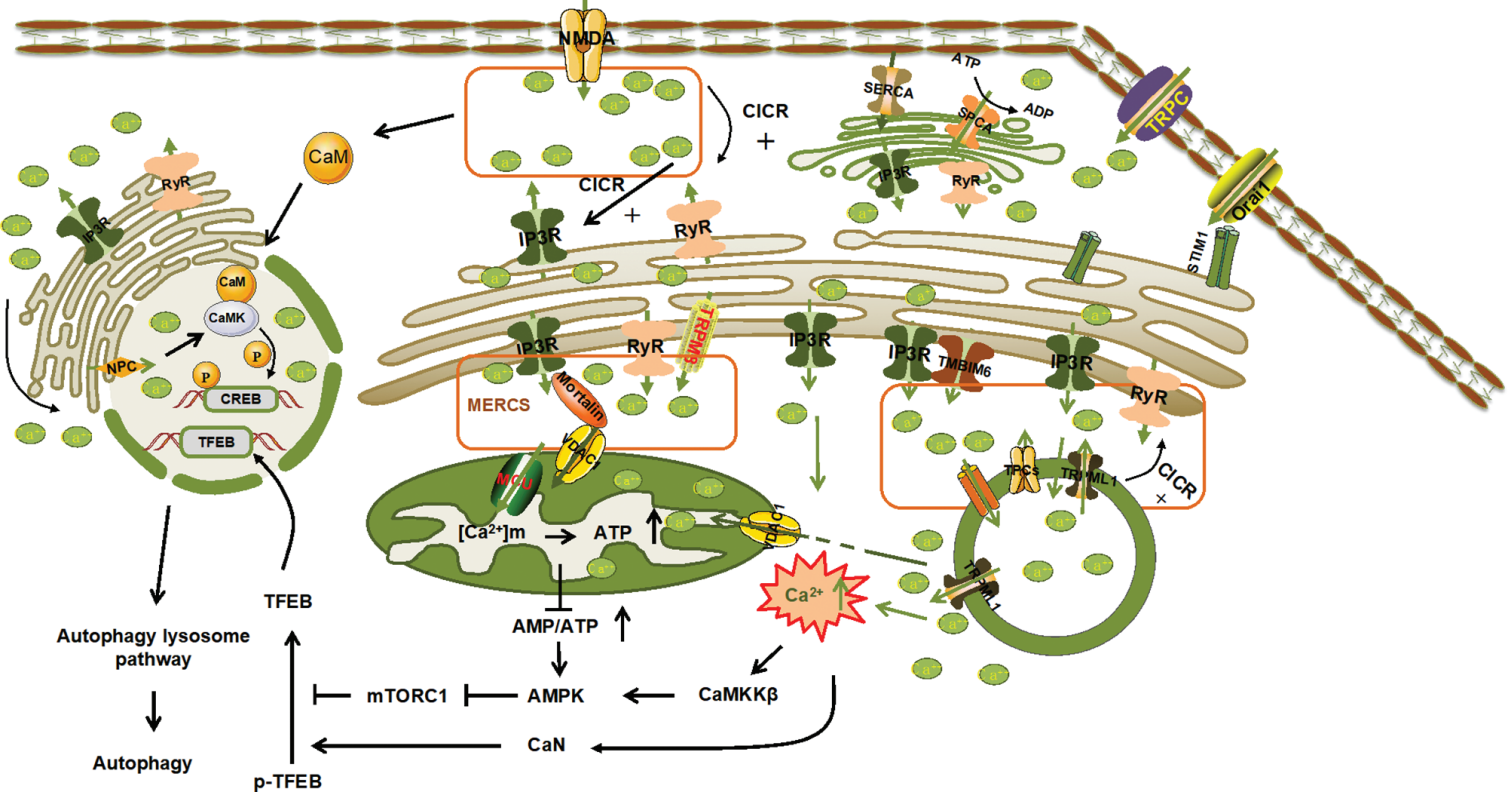
Mechanism diagram of endoplasmic reticulum coordinating with other organelle membranes to regulate Ca^2+^ and thus regulate autophagy. The ER interacts with the cell membrane and other organelles, including mitochondria, lysosomes, and the nucleus, to coordinate Ca^2+^ regulation and influence the initiation and progression of autophagy. AMPK = adenosine 5′-monophosphate (AMP)-activated protein kinase; CaM = calmodulin; CaMKs = Ca^2+^/calmodulin-dependent protein kinases; CaMKKβ = Ca^2+^-calmodulin-dependent kinase kinase β; CaN = calcineurin; CICR = Ca^2+^-induced Ca^2+^ release; CREB = cAMP response element binding protein; IP3 = inositol-1,4,5-triphosphate; IP3R = IP3 receptors; MCU = mitochondrial Ca^2+^ uniporter; MERCS = mitochondria–ER contact sites; mTOR = mammalian target of rapamycin; NMDA = N-methyl-D-aspartate; NPC = nuclear pore complex; Orai1 = Orai calcium Release-Activated calcium Modulator 1; RYR = ryanodine receptor; SERCA = The Sarco ER Ca^2+^ ATPase; SPCA = secretory pathway Ca^2+^-ATPase; STIM1 = stromal interaction molecule; TFEB = transcription factor EB; TMBIM6 = transmembrane BAX inhibitor motif containing 6; TPCs = two-pore Ca^2+^ tunnels; TRPC = transient receptor potential canonical; TRPM8 = transient receptor potential melastatine 8; TRPML1 = transient receptor potential melastatine 1; VDAC1 = the voltage-dependent anion channel 1.

The ER serves as a significant source of lysosomal Ca^2+^, alongside transmembrane BAX inhibitor motif containing 6 (TMBIM6). This TMBIM6, resembling a Ca^2+^ channel, is an integral component of the ER cell membrane. Under conditions of nutrient deprivation or mTOR inhibition, TMBIM6 meticulously regulates the localized release of Ca^2+^ through lysosomal mucolipin 1/transient receptor potential melastatine 1 (TRPML1) channels. This delicate process activates PPP3/CaM phosphoric acid, which in turn triggers transcription factor EB (TFEB) activation, ultimately enhancing autophagy. Therefore, TMBIM6 is recognized as a lysosomal Ca^2+^ regulator that collaborates with autophagy to alleviate metabolic stress. Lysosomal Ca^2+^ is released through TRPML1 and TPC2 plasma channels, initiating the discharge of Ca^2+^ reserves stored within the ER. This intricate mechanism involves the calcium-induced calcium release process. In cases where lysosomal Ca^2+^ reserves are depleted, the ER promptly transports Ca^2+^ to the lysosome via IP3R, ensuring the lysosome is refilled with Ca^2+^.^[31]^ Additionally, lysosomal TRPML1 plays a pivotal role in facilitating the transfer of Ca^2+^ from the lysosome to the mitochondria.^[32]^ These findings underscore the existence of a complex crosstalk among various organelles, collaboratively regulating Ca^2+^ levels. This orchestrated effort subsequently governs the occurrence and development of autophagy (Fig. 2).

### 4.2. Mitochondrial Ca^2+^ regulates autophagy

Mitochondria serve as another major reservoir for Ca^2+^ in the cell, playing a key role in buffering [Ca^2+^]_C_. The accumulation of [Ca^2+^]_m_ stimulates tricarboxylic acid cycle dehydrogenase and promotes oxidative phosphorylation (OXPHOS), ATP and NAD(P)H synthesis, reactive oxygen species generation, mitochondria dynamics, and mitophagy.^[33]^

A low-moderate increase in [Ca^2+^]_m_ is necessary and sufficient for adapting ATP production to meet cellular demands. Under these conditions, autophagy is typically inhibited. However, an overload of [Ca^2+^]_m_ leads to the disruption of mitochondrial membrane integrity, triggers permeability transition, causes irreversible oxidative damage to the mitochondria membrane, and results in a loss of ATP production.^[34]^ This mitochondrial damage initiates mitophagy, a specialized form of autophagy that serves as the main mechanism to counteract mitochondrial damage, to remove the damaged mitochondria.^[33]^ In a study examining cancer cell sensitivity to chemotherapy, it was found that chemotherapy could trigger [Ca^2+^]_m_ overload and inhibit autophagy. This effect counteracts the pro-survival role of autophagy, ultimately leading to apoptosis of cancer cells by activating the apoptosis pathway.^[35]^ To sum up, while a low-moderate increase in [Ca^2+^]_m_ can positively regulate autophagy, the effect of [Ca^2+^]_m_ overload on autophagy remains a subject of controversy.

Transferring Ca^2+^ from the ER to mitochondria via the mitochondrial calcium uniporter (MCU) can increase [Ca^2+^]_m_, which promotes mitochondrial OXPHOS and inhibits SIRT1-dependent autophagy by regulating α-ketoglutarate dehydrogenase activity.^[36]^ MCU, which facilitates mitochondrial Ca^2+^ influx, plays a crucial role in regulating autophagy. Luong et al demonstrated that MCU can reduce [Ca^2+^]_C_ by taking up mitochondrial Ca^2+^, thereby promoting autophagy.^[37]^ Additionally, MCU increases the nuclear translocation of TFEB in breast cancer cells, thereby enhancing autophagy and promoting the migration of these cells.^[38]^

### 4.3. Lysosomal Ca^2+^ regulates autophagy

Lysosomes in living cells are crucial “Ca^2+^ pools,” which play a significant role in maintaining calcium homeostasis and Ca^2+^ signal transduction. Abnormal increases or decreases in lysosome Ca^2+^ levels can lead to lysosomal dysfunction, impairing cellular uptake and excretion dysfunction, and ultimately damaging the autolysosomal pathway. This damage contributes to the occurrence and development of neuronal senescence.^[39]^ Ca^2+^ channels are essential for maintaining lysosomal calcium homeostasis, and alterations in lysosomal pH have also been observed to disrupt this balance.^[40]^ Endolysosomal Ca^2+^ channels primarily include members of the transient receptor potential protein family, such as TRPMLs and transient potential receptor melastatin-2 (TRPM2), as well as two-pore Ca^2+^ tunnels (TPCs). The regulation of these channels is closely linked to autophagy.^[41]^ A loss of TRPML-1 function leads to altered autophagic flux, characterized by increased accumulation of autophagosomes and delayed fusion between autophagosomes and lysosomes.^[42]^ Medina et al proposed that lysosomal Ca^2+^ channel mucolipin 1, also known as TRPML1, facilitates Ca^2+^ release under starvation conditions, causing an increase in [Ca^2+^]_C._ This elevation in [Ca^2+^]_C_ activates calcineurin (CaN) through binding to CaM, which subsequently promotes the nuclear translocation of TFEB and enhances autophagy.^[43]^ The Ca^2+^ release mediated by TRPML1 has also been demonstrated to regulate lysosome motility, promoting the movement of lysosomes towards autophagosomes.^[44]^ The TRPM2 is another Ca^2+^ channel found not only on the plasma membrane but also on the lysosomal membrane.^[45]^ TRPM2 has been identified as a prognostic marker for prostate cancer, and it has the ability to inhibit autophagy, although the specific mechanism remains unclear. In PC-3 cells, silencing of TRPM2 was found to alter the expression of autophagy-related genes and induce autophagy.^[46]^ Similar to TRPML-1, TPCs are also implicated in the regulation of autophagy. Leucine-rich repeat kinase 2 (LRRK2) facilitates the release of lysosomal Ca^2+^ through TCPs and activates downstream CaMKK/AMPK and CaN/TFEB signaling pathways, thus promoting the expression of autophagy-related genes.^[47]^ In summary, lysosomes affect calcium homeostasis primarily by modulating the permeability of Ca^2+^ channels, which in turn activates TFEB and ultimately enhances autophagy.

### 4.4. Plasma membrane Ca^2+^ channels regulate autophagy

Plasma membrane Ca^2+^ channels, crucial for regulating Ca^2+^ influx, have a significant impact on cellular calcium homeostasis.^[48]^ In human melanoma cells, silencing of the T-type Ca^2+^ channel gene disrupts [Ca^2+^]_C_ levels by blocking Ca^2+^ influx, thereby inhibiting autophagy.^[49]^

Orai1, STIM1, and TRPC1 are all involved in the regulation of autophagy. It is well-established that the calcium release-activated calcium channel, primarily composed of Orai1 hexamers, is located in the plasma membrane. STIM1, a Ca^2+^ sensor in the ER membrane, plays a key role in this process. Store-operated Ca^2+^ entry (SOCE) mediated by TRPC1 requires the participation of both Orai1 and STIM1.^[50]^ When [Ca^2+^]_ER_ levels decrease, STIM1 undergoes oligomerization and translocates to the membrane, where it interacts with Orai1 and TRPC1, thereby triggering SOCE.^[51]^ Studies confirmed that activation of Orai1 increased [Ca^2+^]_C_ levels through SOCE, subsequently activating CaN/TFEB and promoting the expression of autophagy-related genes and vacuolization in a mouse model of acute pancreatitis.^[52]^ Sukumaran et al demonstrated that hypoxia and starvation can trigger autophagy and increase cell viability via SOCE, mediated by the upregulation of TRPC1 expression. However, the expression levels of STIM1 and Orai1 remained unchanged.^[53]^ These varying results across different cells and diseases suggest that the dominant Ca^2+^ channel may differ under certain conditions, leading to discrepancies in experimental outcomes.

Several studies have established a connection between voltage-gated calcium channels (VGCCs) and the regulation of autophagy. The application of various VGCC antagonists in different disease contexts can both induce and inhibit autophagy. This variability may arise because the effect of Ca^2+^ channel blockers on autophagy is not solely due to the inhibition of Ca^2+^ channel function but could also involve direct effects on lysosomal function.^[54]^ Therefore, further investigations should focus on assessing the role of different VGCCs in autophagy regulation through channel knockdown or knockout methods, rather than relying exclusively on pharmacological approaches.

The mammalian TRP channel superfamily is divided into 6 subfamilies, with some members serving as plasma membrane Ca^2+^ channels that play important roles in regulating autophagy. Evidence indicates that TRPV1, TRPV2, and TRPV4 channels positively regulate autophagy. TRPC1-mediated Ca^2+^ entry is essential for regulating autophagy in response to hypoxia and nutrient depletion. Upregulation of TRPC4 raises intracellular Ca^2+^ levels, which activates the Ca^2+^-CAMKK2-AMPK pathway, leading to mTOR inhibition and subsequent autophagy. Both TRPC1 and TRPC4 channels act as positive regulators of autophagy. Conversely, the TRPM2 channel is involved in the regulation of oxidative stress-induced autophagy. TRPM2-mediated Ca^2+^ influx functions as an autophagy inhibitor, negatively regulating oxidative stress-induced autophagy. Meanwhile, TRPM3 positively affects autophagy by indirectly controlling autophagosome extension through modulation of LC3 protein expression. Additionally, TRPM7 promotes basal autophagy via the CAMKK2-AMPK pathway.^[54]^ Therefore, plasma membrane Ca^2+^ channels can regulate autophagy levels through different mechanisms.

### 4.5. Ca^2+^ regulates autophagy in other organelles

#### 4.5.1. Nuclear Ca^2+^ regulates autophagy

Nuclear Ca^2+^ has emerged as a crucial mediator in the interaction between synapses and the nucleus, which can regulate transcription factor activity, neuronal gene expression, and more. A reduction in nuclear Ca^2+^ levels is neurotoxic and contributes significantly to the progression of neurodegenerative diseases, also affecting neuronal autophagy. The shared lumen of the ER, nucleoplasmic reticulum, and nuclear membrane represent the most abundant storage of Ca^2+^ and can release ions in response to various molecular triggers. Ca^2+^ enters the nucleus via 2 main pathways: directly from the cytoplasm or from the ER. IP3Rs and RyRs on the outer nuclear membrane play a role in regulating nuclear Ca^2+^ levels. Ca^2+^ ions from the ER can enter the nucleus, for example, through the big potassium channel (BK). The second pathway involves Ca^2+^ entry through nuclear indentations enriched with nuclear pore complexes. IP3R and RyR channels facilitate the flow of Ca^2+^ from the ER to the cytoplasm, while nuclear pore complexes aid in Ca^2+^ transport into the nucleus. An increase in nuclear Ca^2+^ triggers the activation of CaMKII and CaMKIV and the phosphorylation of cAMP response element binding protein, thereby regulating epigenetic and transcriptional processes (Fig. 2).^[55,56]^

#### 4.5.2. Golgi Ca^2+^ regulates autophagy

Over the past 20 years, numerous direct and indirect lines of evidence have suggested that the Golgi apparatus also plays a key role in intracellular Ca^2+^ storage. Similar to ER Ca^2+^ channels, IP3Rs and RyRs are the 2 primary types of Ca^2+^ release channels in the Golgi. Additionally, 2 ATP-dependent Ca^2+^ pumps, Sarco/Endoplasmic Reticulum Ca^2+^ ATPase, and secretory pathway Ca^2+^-ATPase, are responsible for Ca^2+^ uptake. Ca^2+^ binding proteins are also involved in maintaining calcium homeostasis within the Golgi apparatus.^[57,58]^ While there is a well-established connection between the Golgi and ER in Ca^2+^ transport, the impact of Golgi regulation of Ca^2+^ level on autophagy is not well-studied and warrants further investigations.

#### 4.5.3. Autophagic Ca^2+^ channel itself controls autophagy

TRPML3, a member of the transient receptor potential cation channel ML subfamily, is uniquely positioned within the endocytic pathway and mature autophagosomes as the sole Ca^2+^ release channel in autophagosomes. It plays a pivotal role in supplying the essential Ca^2+^ necessary for autophagy-related processes, including the regulation of autophagosome production and lysosomal fusion.^[54,59]^ Research indicates that the presence of TRPML3 within autophagosomes is nearly ubiquitous, and its expression level is directly associated with the enhancement of autophagy. TRPML3 acts as a downstream effector of phosphatidylinositol-3-phosphate, directly and specifically activating TRPML3 on the cytoplasmic and intracellular sides. Proper activation of TRPML3 can counteract the inhibition of autophagy caused by blocking the phosphatidylinositol-3-phosphate upstream signaling pathway.^[60,61]^

## 5. Research progress of Ca^2+^-regulated autophagy in neurodegenerative diseases

### 5.1. Effect of Ca^2+^-regulated autophagy in PD

The pathogenesis of PD is complex and involves multiple factors such as abnormal autophagy, calcium homeostasis imbalances, protein aggregation, and oxidative stress.^[1]^ Of particular interest is the relationship between calcium homeostasis imbalances and abnormal autophagy, which has garnered significant attention in the study of PD.

#### 5.1.1. Plasma membrane Ca^2+^ channels regulate autophagy in PD

It has been confirmed that the abnormal SOCE mediated by TRPC1 contributes to the damage of dopaminergic neurons in PD.^[62]^ In PD patients and animal models treated with dopaminergic neurotoxins 6-hydroxydopamine, 1-methyl-4-phenylpyridinium (MPP^+^), or 1-methyl-4-phenyl-1,2,3,6-tetrahydropyridine, downregulation of TRPC1 and autophagy markers has been observed. Restoring TRPC1 expression can rescue dopaminergic neurons by increasing Ca^2+^ influx to promote autophagy.^[63]^ It remains uncertain whether Orai1 or TRPC-mediated Ca^2+^ influx promotes autophagy in PD, and both may be involved. Cu_2 − *x*_Se-anti-TRPV1 nanoparticles activate the TRPV1 channel on the plasma membrane, allowing Ca^2+^ inflow, which in turn activates the ATG5 and Ca^2+^/CaMKK2/AMPK/mTOR signaling pathways, thereby promoting α-syn phagocytosis and degradation. This can improve motor function in PD mice.^[64]^ VGCCs are the primary source of Ca^2+^ entry into electrically excited cells. In neurons, VGCCs or receptor-mediated channels increase cytoplasmic Ca^2+^ transients. In dopaminergic neurons, activation of L-type VGCC Ca^2+^ channels raises [Ca^2+^]c and activates calpain, which inhibits autophagy.^[65]^ In addition, recent studies have found that the endogenous protein endoophilina-A (EndoA) facilitates Ca^2+^ flow through the membrane into synaptic terminals during neuronal activity, thereby enhancing the structural flexibility of EndoA. This process allows EndoA to translocate from the plasma membrane into the synaptic lumen, where it promotes the formation of autophagosomes. Conversely, Parkinson risk variants hinder its localization to the plasma membrane, thus blocking autophagy induction associated with neuronal activity.^[66,67]^

#### 5.1.2. Lysosome Ca^2+^ regulates autophagy in PD

Recent studies have found that in SH-SY5Y cells with either wild-type α-syn overexpression or α-syn mutations (such as A30P and A53T), lysosomal calcium homeostasis and pH are altered. This leads to decreased expression of lysosomal-associated membrane protein 1 and disruption of lysosomal morphology and distribution, thus inhibiting neuronal autophagy.^[68]^ This evidence suggests that the abnormal accumulation of α-syn in familial early-onset PD may affect lysosomal calcium homeostasis and impede autophagy in dopaminergic neurons. TPCs are lysosomal ion channels involved in Ca^2+^ signal transduction.^[69]^ They can be activated by LRRK2 and regulate autophagy through lysosomal Ca^2+^-mediated events.^[70]^ Mutations of LRRK2 can lead to advanced PD, and its overexpression can activate TPC2, resulting in substantial mobilization of lysosomal Ca^2+^ and subsequent efflux of [Ca^2+^]_ER_. This activation leads to the Ca^2+^/CaMKKβ/AMPK pathway being triggered, which promotes a continuous increase in autophagosome formation.^[71]^ Therefore, in advanced PD cases with LRRK2 deficiency, enhancing LRRK2 expression to boost Ca^2+^-dependent autophagy may represent a novel therapeutic approach for PD. Additionally, TRPML1, another important Ca^2+^ channel in lysosomes, promotes the autophagosome maturation. In neurons, TRPML1 antagonist ML-SI3 impedes the clearance of autophagosomes, while TRPML1 agonist ML-SA1 increases the abundance of autophagosomal vesicles containing α-syn. However, the effect of ML-SA1 on α-syn aggregates is also influenced by increased [Ca^2+^]_C_.^[72]^

#### 5.1.3. Mitochondria Ca^2+^ regulates autophagy in PD

In PD, mitochondria have also been identified as important Ca^2+^ storage compartments. MCU, located in the mitochondria membrane, plays a critical role in calcium homeostasis by promoting the uptake of [Ca^2+^]_C_, which in turn regulates autophagy. MPP^+^, a neurotoxin associated with PD, inhibits MCU expression and disrupts mitochondrial OXPHOS by lowering [Ca^2+^]_m_. This results in an increased AMP/ATP ratio, activation of AMPK, and subsequent induction of ACD. Similarly, the knockout of MCU can lead to excessive autophagy through AMPK pathway activation. Treatment with MCU agonists has been shown to mitigate MPP^+^-induced excessive autophagy and improve cell viability.^[73]^ This suggests that down-regulation of MCU may participate in the pathogenesis of PD by reducing [Ca^2+^]_m_ and inducing pathological autophagy. Additionally, disturbances in the ER-mitochondrial signaling pathway have been observed in PD, affecting calcium homeostasis and autophagy. However, the precise mechanisms by which calcium homeostasis regulates autophagy in this context remain unclear.

### 5.2. Effect of Ca^2+^-regulated autophagy in AD

AD is the most prevalent form of dementia, characterized by protein misfolding, intracellular tau tangles, and extracellular Aβ plaque accumulation. The imbalance of calcium homeostasis is believed to play a significant role in the development of AD.^[74]^ In various AD models, abnormalities in calcium homeostasis in the cytoplasm, ER, and mitochondria are closely linked to impaired autophagy.

#### 5.2.1. Endoplasmic reticulum Ca^2+^ regulates autophagy in AD

Familial Alzheimer disease (FAD) follows Mendelian inheritance patterns and is primarily associated with mutations in the APP, PS1, and PS2 genes. Research into the mechanisms underlying FAD has shown that PS2 mutations can induce depletion of [Ca^2+^]_ER_ in different FAD-PS2 cell models. This depletion impairs [Ca^2+^]_C_ response to IP3-linked cell stimulations and disrupts autophagosome-lysosome fusion by reducing the recruitment of the small GTPase RAB7 to autophagosomes, ultimately contributing to the development of FAD.^[75]^ Furthermore, studies using APP/PS1 double transgenic mice and SH-SY5Y cells treated with Aβ have demonstrated that Aβ regulates autophagy through the receptor for advanced glycation end products (RAGE)/Ca^2+^/CaMKKβ/AMPK pathway. This regulation promotes the deposition of pathological autophagic vesicles and the formation of autophagosomes. These pathological changes can be mitigated by using BAPTA-AM, AMPK inhibitors, or through genetic interference.^[76]^

Another study in APP/PS1 double transgenic mice demonstrated that YZFDF could inhibit autophagy by inhibiting the RAGE/CaMKKβ/AMPK/mTOR pathways. This intervention improved both the pathological deposition of Aβ and cognitive impairments in the AD model.^[77]^ These findings, along with the previous studies, underscore the crucial role of the Ca^2+^/CaMKKβ/AMPK pathway in Aβ-induced autophagosome formation in AD. In addition, RyR3-mediated ER Ca^2+^ regulation has been shown to influence autophagy and programmed cell death in neural stem cells. In I (−) RYR3KO hippocampal neurons, the effects of ER Ca^2+^ are significantly diminished. Conversely, in an insulin withdrawal model, RyR3 expression and activation are increased, leading to elevated ER Ca^2+^ release. This rise in cytoplasmic Ca^2+^ subsequently triggers a signaling cascade that induces ACD in hippocampal neurons.^[21]^ Furthermore, increased RyR2 activity in AD may inhibit neuronal autophagy and contribute to Aβ accumulation. Basal RyR2 activity regulates autophagy through modulation of the CaN/p-AMPK/p-ULK1 pathway.^[78,79]^

#### 5.2.2. Mitochondria Ca^2+^ regulates autophagy in AD

Cognitive decline in AD can be exacerbated by mitochondrial dysfunction resulting from disrupted mitochondrial calcium homeostasis. MCU is a key channel for mitochondrial Ca^2+^ uptake and may be a potential target for AD therapy. In AD mouse models, knockdown of MCU has been shown to enhance memory performance, and this intervention leds to an increase in Beclin-1 levels and a decrease in P62 levels.^[80]^ In addition, an imbalance in calcium homeostasis can affect mitochondrial function, thereby impairing mitophagy, which play a role in AD’s development.^[81]^

#### 5.2.3. Lysosomal Ca^2+^ regulates autophagy in AD

In AD, lysosomal pH and Ca^2+^ levels are critical in regulating neuronal autophagy. Aβ can increase lysosomal pH and decrease lysosomal Ca^2+^ concentration, leading to abnormal autophagy and neuronal death.^[39]^ Using the TPC/nicotinate adenine dinucleotide phosphate (NAADP) antagonist Ned19 or tetrandrine, or blocking TPC2 hyperactivity by knocking down TPC2, has been shown to restore lysosomal Ca^2+^ levels and pH, thereby normalizing autophagy.^[82,83]^ Some researches have confirmed that abnormal TRPML1 activity can disrupt lysosomal Ca^2+^ concentration, contributing to AD pathogenesis by affecting autophagy, at least partially through the PPARγ/AMPK/mTOR signaling pathway.^[84]^ In late-onset AD iPSC models, reduced TRPML1-mediated lysosomal Ca^2+^ release has been observed, while the TRPML1 agonist ML-SA1 can rescue alterations in the endocytosomal-autophagolysosomal system associated with AD.^[85]^ Additionally, PS1 deletion or AD-related mutations can impair lysosomal acidification and proteolysis, thereby inhibiting autophagy. PS1 knockout cells exhibit increased lysosomal pH, abnormal TRPML1-mediated lysosomal Ca^2+^ efflux, and elevated cytoplasmic Ca^2+^, all of which contribute to autophagy inhibition.^[86]^ Abnormal intracellular Ca^2+^ signaling in AD disrupts lysosomal Ca^2+^ release through TRPML, impairs autophagosome biosynthesis, affects synaptic plasticity, and results in the deposition of Aβ42 and P-tau.^[87]^

#### 5.2.4. Plasma membrane Ca^2+^ regulates autophagy in AD

In 3xTg AD mouse models, activation of the Ca^2+^ permeable channel TRPV1 attenuated memory deficits and reduced the accumulation of Aβ and phosphorylated tau proteins.^[88]^ It is well-established that overactivation of NMDARs is a key factor in increasing intracellular Ca^2+^ levels, which leads to synaptic dysfunction and neuron loss. NMDA receptor antagonists mitigate Aβ deposition by regulating the calpain-1 signaling pathway and autophagy, thereby improving cognitive dysfunction in 5xFAD mice. Additionally, oral NMDAR inhibitors can reduce anxiety-like behavior and enhance autophagy flux in 5xFAD mice.^[89]^ In AD, intracellular Ca^2+^ can inhibit autophagy flux by increasing Orai1, which suppresses the activation of AKT and mTOR. Intracellular Ca^2+^ can also enhance the CaMKKβ/AMPK/mTOR pathway through TRPC, further inhibiting autophagy flux.^[78]^ As a typical Ca^2+^ transporter on cell membranes, APOE4 exacerbates the effect of Aβ on lysosomal membrane instability and permeability, resulting in impaired autophagy and lysosomal degradation in N2a cells.^[90]^

### 5.3. Effect of Ca^2+^-regulated autophagy in ALS

#### 5.3.1. Lysosome Ca^2+^ regulates autophagy in ALS

Abnormal autophagy resulting from disrupted lysosomal calcium homeostasis is considered highly significant in the pathogenesis of ALS.^[12]^ In a neuronal model of the ALS/Parkinson-Dementia complex, established by exposure to the neurotoxin β-methylamino-L-alanine (L-BMAA), it was observed that TRPML1 expression was decreased, leading to impaired calcium homeostasis in both lysosomes and the ER. In contrast, ML-SA1, a TRPML1 agonist, was found to induce the release of lysosomal Ca^2+^ in a dose-dependent manner, thus reducing the accumulation of autophagy-related proteins p62/SQSTM1 and LC3-II, which are elevated by L-BMAA.^[91]^ These findings demonstrate that alterations in [Ca^2+^]_C_ homeostasis induced by TRPML1 activation can promote autophagy, ultimately rescuing ALS motor neurons.

#### 5.3.2. Endoplasmic reticulum Ca^2+^ regulates autophagy in ALS

The mutation in the ER chaperone sigma-1 receptor (SigR1) is one potential cause of ALS.^[92]^ In NSC-34 motor neurons, SigR1 depletion can lead to Ca^2+^ mobilization, mitochondrial and ER defects, impaired autophagy degradation, and changes in endosomal traffic.^[93]^ The SigR1-E102Q mutation, in particular, is associated with ALS in adolescents. This mutant protein accumulates rapidly in the ER and related compartments in transfected cells, triggering structural changes in the ER, nuclear membrane, and mitochondria, and leading to disrupted calcium homeostasis. Additionally, ER defects and proteotoxic stress generated by E102Q-SigR1 aggregation further contribute to autophagy impairment.^[94]^ However, it remains unconfirmed whether the defect in calcium homeostasis caused by the SigR1-E102Q mutation directly causes the impairment of autophagy degradation in motor neurons.

#### 5.3.3. Plasma membrane Ca^2+^ regulates autophagy in ALS

In the spinal cord of the SOD1G93A mouse model of ALS, verapamil, a plasma membrane Ca^2+^ channel blocker, can inhibit the activation of calpain-1. Calpain-1 is a Ca^2+^-dependent cysteine protease that cleaves various autophagy-related proteins, including Atg5, and disrupts the formation of the Atg12-Atg5 complex, thereby inhibiting autophagy. By blocking Ca^2+^ influx, verapamil promotes autophagy flow and helps protect motor neurons.^[95]^

### 5.4. Effect of Ca^2+^-regulated autophagy in other diseases

HD is a late-onset autosomal dominant neurodegenerative disorder characterized by progressive motor abnormalities and cognitive deficits. The disease is caused by mutations in the Htt gene, leading to the accumulation of mutant Htt (mHtt) protein. This accumulation can induce ER stress, disrupt calcium homeostasis, and impair autophagy.^[96]^ Sarkar concluded that mHtt can increase extracellular Ca^2+^ influx and bind to IP3R, sensitizing the Ca^2+^ channels. This results in the release of [Ca^2+^]_ER_, ultimately causing an imbalance in [Ca^2+^]_C_ homeostasis and inhibiting autophagy activity.^[97]^ In HD, Williams et al confirmed that increased [Ca^2+^]_C_ can cause intracellular excitotoxicity and inhibit autophagy, thereby exacerbating the pathological damage caused by mHtt. The use of the L-type Ca^2+^ channel blocker verapamil has been shown to restore these changes.^[98]^ This effect may be related to the inactivation of calpain-1. It has been demonstrated that overexpression of calpain inhibitors, such as calpastatin, increases autophagosome levels and provides protective effects in HD mouse models, improving motor signs and delaying the onset of tremors.^[99]^ Recent studies have also revealed that NAADP is an intracellular second messenger that promotes Ca^2+^ release through the activation of TPCs in the endosomal system. Overexpression of mHtt-Q74 leads to increased NAADP-induced Ca^2+^ signaling and enhanced mHtt aggregation. The administration of the TPC antagonist Ned-19 or the Ca^2+^ chelator BAPTA-AM has been shown to reduce mHtt aggregation, while silencing TPC2 restores mHtt aggregation. This suggests that regulating mHtt aggregation and autophagy by lysosomal calcium homeostasis could serve as a potential neuroprotective mechanism.^[100]^ However, recent years have seen limited research on the regulation of autophagy by Ca^2+^ in HD. As a result, the notion that “Ca^2+^ inhibits autophagy” has not been widely accepted and applied in other HD-related studies.

Spinal muscular atrophy (SMA) is a group of motor neuron diseases characterized by muscle weakness and atrophy. Motor neurons, which originate in the spinal cord, are affected in SMA. In SMA, ERK MAPK inhibits mTOR phosphorylation and reduces autophagy markers in human SMA spinal motor neurons. Additionally, the calcium chelator BAPTA-AM inhibits ERK phosphorylation and decreases LC3-II levels.^[101]^

Lysosomal storage disorders (LSDs) constitute a group of neurodegenerative diseases marked by the accumulation of lysosomal substrates. Multiple studies have revealed defects in calcium homeostasis and autophagy associated with these disorders, suggesting a potential causal relationship between the two. BK, a well-known potassium channel, is also expressed on lysosomal membranes. Dysfunction of BK leads to compromised lysosomal Ca^2+^ signaling and abnormal lipid accumulation, which is a hallmark phenotype of most LSDs. Enhancing BK activity has been found to alleviate lipid accumulation in several LSD cell models,^[102]^ indicating that impaired lysosomal Ca^2+^ signaling can cause abnormal degradation of lysosomal substrates and subsequent lipid accumulation. Di Paola S summarized previous studies and concluded that mutations in the gene encoding TRPML1 alter lysosomal calcium homeostasis and contribute to mucolipidosis type IV, a recessive form of LSD. At the cellular level, fibroblasts from mucolipidosis type IV patients exhibit enlarged and engulfed late endo-lysosomal compartments, impaired autophagy, and the deposition of lipids and glycosaminoglycans.^[103]^ This evidence further supports the involvement of abnormal autophagy, triggered by altered lysosomal Ca^2+^ signaling, in the pathogenesis of LSDs. In summary, impaired Ca^2+^ signal transduction causing abnormal lysosomal degradation is a key pathogenic mechanism in LSDs, which leads to subsequent pathological changes.

## 6. Conclusions and prospects

In summary, Ca^2+^ and autophagy cannot be regarded as absolute “good” or “bad” in the context of neurodegenerative diseases, and their relationship is more complex than previously understood. This relationship is influenced by factors such as the cell’s energy state, stress levels, and the interplay of signaling pathways between organelles. What is the mechanism of intracellular calcium homeostasis imbalance regulating autophagy? Disruptions in Ca^2+^ storage compartments and Ca^2+^ channels affect calcium homeostasis and, consequently, impact autophagy. As a crucial second messenger, Ca^2+^ can either promote or inhibit autophagy by modulating the activity of various intracellular signaling pathways, such as the Ca^2+^/CaMKKβ/AMPK pathway, the CaN/mTOR/TFEB pathway, the ERK pathway, and the PPP3/CaN/TFEB pathway. These insights only scratch the surface of a more intricate network. Are there additional Ca^2+^ channels in various Ca^2+^ storage compartments that regulate autophagy? Are other pathways involved? Can effective drugs be developed targeting these pathways in the future? These questions remain unanswered. Completing the understanding of the intracellular network of Ca^2+^-regulated autophagy is crucial for guiding future research and the development of new therapeutic strategies.

## Author contributions

**Resources:** Li Wang.

**Supervision:** Zhixiao Zhang, Zexin Fan, Lei Huang, Li Wang.

**Writing – original draft:** Meng Hou.

**Writing – review & editing:** Meng Hou.
